# Curious Case of Ovarian Gestation

**Published:** 1803-01-01

**Authors:** 


### *Curious Case of Ovarian Gestation; communicated by our Correspondent at Paris.*

THE subject of the observation was brought from the Hotel Dieu to the dissecting room of the Medical School; but owing to the industry of the student and reporter of the case, some of the circumstances of the life of the person have been ascertained, which tend to render the account still more curious and interesting. Among a number of subjects brought for dissection, was a girl of thirteen years of age, and remarkable for the extraordinary state of emaciation of her body, the leaden colour of the skin, the sunken eyes, and the elevation of the zygomatic arches. The breasts were not the least develloped, and the abdomen not at all raised, or any appearance of fullness. The mons veneris was unfurnished, the labia externa hardly existed, but the clitoris was very large, and the hymen, which was perfect, hardly admitted, notwithstanding the fluid state of the parts, of the introduction of the point of the little finger. On opening the body, all the abdominal viscera were found to adhere. At the left side of the cavity was a pouch contracting in the middle, so as to appear like two lobes. This pouch or cyst, resting on the brim of the pelvis, thrust upwards the intestines, and pressed on the ureter, which was large, and filled with urine as well as the pelvis of the kidney. The great diameter of this tumour was about eight inches, and its smaller about five or six; on its outer surface was an opening or communication between the cyst and the cavity of the abdomen; both cavities contained a purulent liquor of a greenish yellow colour. Corresponding to the exterior contraction, a membrane was thrown across internally, which divided the pouch into two cavities, that communicated by means of a considerably large opening; in the inferior of these, besides some hair, was found a quantity of fat, and the coronæ of teeth, cartilages, and pieces of bones, both long and flat, but without any precise form to distinguish them; there was, however, a piece of the lower jaw, in which were found the coronæ of the molar and canine teeth, and beneath these was perceivable the gelatinous substances intended to form the fangs. This tumour was connected superiorly by a narrow stalk to that part of the lateral ligaments, where the ovarium is usually found, but in this instance, it had totally disappeared on that side; the Fallopian tube was perfect, and ascended somewhat on the the side of the tumour. The matrix, which was in its natural situation, was uncommonly small, and the right ovarium, somewhat enlarged, had attached to it small excresences of the size of peas. The contents of the thorax were as usual, except the left lung, which was contracted, of a blackish colour, and solid consistence.

The reporter of this case has been able to ascertain the following facts. Her name, Louis Adelaide D***, was born at Versailles, May 1, 1789; her father, who was a taylor, in poor circumstances, had placed her at the age of five years at the Salpetriere, which she left at ten, to live in Paris with her father till she arrived at her eleventh year, when she was employed by a dealer to run of errands, and which she continued to do six months. She quitted this employment, and was placed with an innkeeper, where a number of journeymen taylors lived, and where she was occupied in the discharge of the meanest offices in the kitchen; from hence she lived as servant to a taylor, where she remained two months, and then apprenticed herself to a bookbinder, whose wife surprized her repeatedly in the act of masturbation, a habit which she acknowledged to have contracted while in the Salpetriere. Her health at last diminishing, she was admitted the 22d Pluviose last, into the Magdalen ward of the Hotel Dieu, where she was treated for that kind of consumption attributed to masturbation. She died the 8th Prairial, without the state of the ovarium being ever suspected.

Many circumstances, not one of which considered isolatedly can be looked on either as rare or extraordinary, concur to render this case interesting. 1st, The youth of the girl. 2d. The absence of menstruation. 3dly, The existence of the hymen and the narrowness of the vagina; and, 4thly, The want of devellopement of the genitals, the clitoris excepted.

*Paris, Nov.* 26, 1802.

